# Effect of Tribocorrosion on Mechanical Behavior of Titanium Dental Implants: An In Vitro Study

**DOI:** 10.3390/ma18051136

**Published:** 2025-03-03

**Authors:** Erika Vegas-Bustamante, Gemma Sanmartí-García, Javier Gil, Luis Delgado-Garoña, Rui Figueiredo, Octavi Camps-Font, Mª Ángeles Sánchez-Garcés, Jorge Toledano-Serrabona

**Affiliations:** 1 School of Medicine and Health Sciences, Campus de Bellvitge, University of Barcelona, C/Feixa Llarga, s/n, Pavelló Govern, 2ª Planta, Despatx 2.9, 08907 L’Hospitalet de Lllobregat, Spain; erikavegasbustamante@ub.edu (E.V.-B.); gemmasanmarti@ub.edu (G.S.-G.); ruibarbosa@ub.edu (R.F.); masanchezg@ub.edu (M.Á.S.-G.); jtoledano@ub.edu (J.T.-S.); 2 Dental and Maxillofacial Pathology and Therapeutics Research Group, IDIBELL Research Institute, 08908 Barcelona, Spain; 3 Bionspired Oral Biomaterials Interfaces, Departament Ciencia e Ingenieria de Materiales, Escola d’Enginyeria Barcelona Est, Universitat Politecnica de Catalunya, Av. Eduard Maristany 16, 08019 Barcelona, Spain

**Keywords:** dental implant, peri-implantitis, implantoplasty, corrosion, Ti6Al4V

## Abstract

Background/Objectives: Peri-implantitis often necessitates surgical intervention, with implantoplasty being proposed as a decontamination method in resective surgeries. This mechanical cleaning technique aims to halt disease progression by removing bacterial colonies. However, implantoplasty may compromise mechanical properties, reduce corrosion resistance, and lead to cytotoxic effects due to titanium particle release. This study aimed to evaluate the corrosion and mechanical resistance of implantoplasty-treated dental implants, with and without bacterial contamination. Methods: Twenty dental implants were divided into three groups: control (C), implantoplasty (IP), and implantoplasty with bacterial contamination (IPC) using *Streptococcus aureus* and *Porphyromonas gingivalis*. Scanning electron microscopy was used to assess surface morphology. Fatigue life curves were obtained using a Bionix servohydraulic machine, and electrochemical corrosion tests were conducted to measure corrosion potentials and intensities. Results: The IPC group demonstrated significantly lower fatigue resistance and higher susceptibility to corrosion compared to the control and IP groups. Fatigue life decreased by 21.7%, and corrosion current density (ICORR) increased from 0.025 μA/cm^2^ (control) to 0.089 μA/cm^2^ (IP) and 0.122 μA/cm^2^ (IPC). Corrosion potential (ECORR) shifted from −380 mV (control) to −450 mV (IP) and −495 mV (IPC). Surface defects caused by bacterial colonization facilitated stress concentration and crack initiation during fatigue testing. Conclusions: Dental implants treated with implantoplasty and exposed to bacterial contamination exhibit significantly reduced mechanical and corrosion resistance. Bacterial activity exacerbates surface vulnerability, leading to titanium loss and pitting corrosion. These findings highlight the clinical implications of bacterial colonization on implantoplasty-treated surfaces.

## 1. Introduction

Titanium (Ti) and its alloys exhibit excellent biocompatibility and corrosion resistance. The amorphous oxide layer formed on the surface of Ti after exposure to air or atmospheric water vapor confers these properties [1]. However, in the oral cavity, this passivation layer can be compromised by mechanical wear (e.g., mechanical decontamination of the implant), electrochemical processes, or combined phenomena (tribocorrosion) [2,3]. These degradation phenomena may affect not only the biocompatibility of the material but also its mechanical properties.

Like any other material, dental implants and their prosthetic components have a limit of resistance beyond which their mechanical properties deteriorate or even fracture [4,5]. Technical and mechanical complications can occur because of metal fatigue and stress from chewing and, occasionally, from the presence of parafunctional habits [6].

Generally, fatigue mechanisms involve lower loads than static loads; however, they are often the most frequent cause of mechanical failure in dental biomaterials [7,8].

Other factors that can affect the mechanical strength of dental implants include the material (commercially pure titanium or alloy), the implant-prosthetic connection, the implant diameter, the crown-to-implant ratio, and the level of clinical insertion of the implant.

In addition, it has been shown that various oral pathogens can degrade the exposed titanium surface and, consequently, affect its mechanical strength [9,10,11,12], causing pitting corrosion that leads to a significant deterioration of the mechanical properties of the implant [2,9,13].

In the treatment of periimplantitis, there are mechanical methods of decontamination that are aggressive to the dental implant surface. Among these methods, implantoplasty allows the removal of bacterial plaque and the smoothing of the implant surface [14,15,16,17]. However, despite these benefits, disinfection is not complete, and bacterial recolonisation occurs when the surface is exposed to the oral cavity [18].

Several articles, including some in vitro studies, have investigated the mechanical resistance of dental implants after implantoplasty [7,19,20,21,22,23] and the release of metallic particles with cytotoxic and inflammatory effects on adjacent tissues [24,25].

Lozano et al. [7] reports that the machining of dental implants causes high residual stress on the implant surface, which promotes electrochemical corrosion. Additionally, the particles released into the medium have higher stored energy and, therefore, greater susceptibility to corrosion. Camps-Font et al. [19] observed a decrease in corrosion resistance. The corrosion current density increased from 0.019 A/cm^2^ for the implant as received to 0.069 A/cm^2^ for the smoothed-rough dental implant. Regarding fatigue resistance, there are few studies on implantoplasty-treated implants. The study by Camps-Font et al. [19] suggests that implantoplasty (IP) does not significantly reduce fatigue resistance, whereas Fonseca et al. [26] reported a significant decrease due to this procedure.

However, to the authors’ knowledge, no studies have investigated the effect of bacteria on a surface exposed to implantoplasty. The objective of this research was to evaluate the effect of tribocorrosion, caused by both mechanical degradation (implantoplasty) and bacterial attack, on the surface and mechanical properties of titanium implants.

## 2. Materials and Methods

An in vitro study was conducted using 45 dental implants with a diameter of 4.5 mm and a length of 13 mm with an internal hexagonal connection (Ocean E.C., Avinent Implant System S.L., Santpedor, Spain). These implants were manufactured from a titanium alloy (Ti6Al4V) and underwent a surface treatment combining grit blasting and chemical plasma anodisation (Biomimetic^®^). A total of 36 implants were used for fatigue tests, divided into three experimental groups of 12 implants each. Nine implants were used for corrosion tests, organised into three experimental groups with three implants per group. The first group, referred to as the control group (C), did not receive any treatment. The second group (IP) underwent an implantoplasty protocol. Lastly, the third group (IPC) underwent implantoplasty followed by bacterial culture with strains of *Streptococcus aureus* and *Porphyromonas gingivalis*.

### 2.1. Implantoplasty Procedure

The implantoplasty procedure was performed by a single operator (EVB), modifying the surface of the 6 mm coronal portion of the dental implant [22]. The procedure was carried out using a GENTLEsilence LUX 8000B turbine (KaVo Dental GmbH, Biberach an der Riß, Germany) with constant irrigation. The surface was sequentially modified using a fine-grain tungsten carbide bur (ISO 514, reference H379.314.014, KOMET GmbH & Co. KG, Lemgo, Germany), followed by a coarse-grain silicone polisher (reference 9608.314.030, KOMET GmbH & Co. KG, Lemgo, Germany) and a fine-grain silicone polisher (reference 9618.314.030, KOMET GmbH & Co. KG, Lemgo, Germany), following the protocol proposed by Costa-Berenguer et al. [22]. A new set of burs and polishers was used for each implant. After implantoplasty, the implants were cleaned with distilled water and dried with compressed air.

### 2.2. Bacterial Culture

All implants were sterilised using UV light and washed with phosphate-buffered saline (PBS). They were then transferred to a 24-well microtitration plate. Bacterial culture was performed only on the IPC group. This study utilised two bacterial species: Staphylococcus aureus and *Porphyromonas gingivalis*, given their prevalence in the peri-implantitis microbiome and their role in inflammatory response and bone loss around implants [27]. Different inocula were prepared by adding 100 µL of each bacterium to 5 mL of Brain Heart Infusion (BHI) medium. The flasks were incubated overnight at 37 °C to promote bacterial growth. After incubation, the inocula were diluted to obtain an optical density of 0.1 for each bacterium. Additionally, 1 mL of bacterial suspension was added to each sample in the 24-well plate, and the samples were incubated overnight at 37 °C [28]. Cultures of Staphylococcus aureus were conducted under aerobic conditions, while those of *Porphyromonas gingivalis* were under anaerobic conditions. Scanning electron microscopy was used to observe bacterial adherence. After culturing, the samples were washed with PBS and transferred to a new 24-well plate. Then, 1 mL of 2.5% glutaraldehyde was added to each well, and the samples were refrigerated for 10–15 min. Subsequently, the samples were washed with PBS and dehydrated with increasing concentrations of ethanol (50%, 70%, 90%, and 100%). Finally, the implants were stored in a desiccator.

### 2.3. Corrosion Assays

Each group of samples was individually immersed in a constant volume of electrolyte for all measurements. Hank’s solution (ThermoFisher, Madrid, Spain) was selected as the electrolyte for all tests. The electrolyte’s pH was maintained constant at 6.7 throughout the experiments and was completely renewed for each test. The tests were performed using a PARSTAT 2273 potentiostat (Princeton, San Jose, CA, USA), controlled by Voltamaster 4 software (Radiometer Analytical, Villeurbanne Cedex, France). For open-circuit potential (EOCP) tests and potentiodynamic tests, a saturated calomel reference electrode (KCl) with a potential of 0.241 V relative to the standard hydrogen electrode was used. All tests were conducted at 37 °C in a Faraday cage to eliminate external electrical or electromagnetic fields. Open-circuit potential (EOCP) tests were performed for 5 h on all samples, with measurements taken every 10 s. Potential stabilisation was considered achieved when the variation was less than 2 mV over 30 min, according to ASTM G5 and ISO 10993-5:2009 standards [29,30].

Potentiodynamic cyclic polarisation curves were obtained following ASTM G5 standards. The parameters studied included corrosion intensity (ICORR (μA/cm^2^)) and corrosion potential (ECORR (mV)).

### 2.4. Fatigue Tests

The fatigue tests aimed to determine the stress level at which the samples could endure a total of 5 × 10^6^ cycles, which is considered the fatigue limit, in accordance with the specifications of the international standard ISO 14801:2016 (Dentistry–Implants–Dynamic loading test for endosseous dental implants) [31]. Except for the defect size (6 mm instead of 3 ± 0.5 mm) and the number of cycles (10 × 10^6^ cycles instead of the minimum of 5 × 10^6^ cycles), all specifications were met.

All implants were fixed in identical positions within standardised epoxy resin moulds with a Young’s modulus of elasticity ≥ 3 GPa (EA 3471 A and B Loctite^®^, Henkel AG & Company, Düsseldorf, Germany), ensuring that 6 mm of the intraosseous portion extended beyond the specimen, as this is the implantoplasty-treated area. The load centre was positioned 14 mm above the resin. Identical hemispherical load elements were designed and screwed onto each implant using the manufacturer’s recommended torque of 35 N·cm.

The tests were conducted at room temperature using a servo-hydraulic MTS Bionix 370 Load Frame machine (MTS^®^, Eden Prairie, MN, USA) equipped with a 15 kN load cell (MTS Load Cell 661.19H-03, MTS^®^, Eden Prairie, MN, USA). The sinusoidal load frequency was maintained at 15 Hz. All samples were secured in a stainless steel clamp so that the longitudinal axis of the implant formed a 30° angle with respect to the direction of the applied forces. Each sample was subjected to a maximum of 10 × 10^6^ uniaxial load cycles, perpendicular to the surface of the hemispherical element, with a load range oscillating between a nominal maximum value and 10% of that value (R = 0.1).

These tests were monitored, and data were recorded in real time using TestStar II^®^ software version 5.1 (MTS^®^, Eden Prairie, MN, USA). The tests began with a failure load of 800 N. Subsequently, the load was halved to 400 N, and further tests were conducted consecutively at different loads until a limit of 10 million cycles was reached. When two consecutive samples endured 10 × 10^6^ cycles without failure, an additional test was performed on a third sample.

The number of cycles and the status of each tested sample were recorded. Failure was defined as the moment the material reached its elastic limit or experienced permanent deformation, the implant assembly loosened, or the sample fractured. The results of the fatigue tests were presented in a graph, representing the number of load cycles endured by each sample and the corresponding maximum load.

### 2.5. Statistical Analysis

The obtained data were entered into a Microsoft Excel spreadsheet (Microsoft^®^, Redmond, Washington, DC, USA) and subsequently processed using Stata 14 software (StataCorp^®^, College Station, TX, USA). The normality of the variables was assessed using the Shapiro–Wilk test and a visual analysis of P-P normal plots. In addition, analysis of variance (ANOVA) tables were calculated. In all cases, the required level of statistical significance was set at *p* ≤ 0.05.

## 3. Results

### 3.1. Corrosion Assays

Table 1 presents the results of corrosion resistance studies, including open-circuit potential and potentiodynamic tests. These results can be explained through fundamental corrosion principles and the interaction of materials with their environment.

In our study, the C group presented the highest EOCP (−137.7 ± 5.2 mV), suggesting greater electrochemical stability and a lower corrosion susceptibility. The IP group had a more negative value (−194.3 ± 3.6 mV), indicating a slight decrease in material stability due to the surface treatment by implantoplasty (*p* < 0.001). Finally, the IPC group presented the lowest value (−198.23 ± 5.7 mV), indicating a slight decrease in stability due to implantoplasty (*p* < 0.001). Lastly, the IPC group showed the lowest EOCP (−198.23 ± 5.7 mV), suggesting increased corrosion susceptibility, likely due to implantoplasty and degradation of the TiO_2_ passive layer induced by bacterial contamination. However, the difference between the IP and IPC groups was not statistically significant (*p* > 0.05).

Regarding corrosion current density (ICORR), this parameter indicates the rate of corrosion on a metallic surface. Higher ICORR values correspond to increased corrosion rates and lower corrosion resistance. The results showed that the C group exhibited the lowest values (0.025 ± 0.011 μA/cm^2^), indicating a more stable passive state and lower corrosion rate, consistent with its more positive EOCP value. The IP group displayed a higher corrosion current density (0.089 ± 0.022 μA/cm^2^), suggesting an increased corrosion rate due to surface modifications from implantoplasty. Finally, the IPC group had the highest ICORR (0.122 ± 0.030 μA/cm^2^), indicating that implantoplasty combined with bacterial contamination exacerbates corrosion. These findings suggest that bacterial presence may accelerate corrosion through redox reactions and metabolic byproducts that further degrade the surface. All comparisons between the three groups were statistically significant (*p* < 0.05).

Corrosion potential (ECORR) refers to the equilibrium potential of a metallic surface with its environment, where oxidation and reduction rates are balanced. More negative ECORR values indicate a higher susceptibility to active corrosion. Our results show that the C group had the most positive ECORR (−380 ± 18 mV), indicating better passive stability and higher corrosion resistance. A more positive ECORR suggests that titanium maintains an intact and active TiO_2_ oxide layer, preventing corrosion. The IP group exhibited a more negative ECORR (−450 ± 40 mV), reflecting reduced corrosion resistance due to implantoplasty, which may have altered the passive layer’s integrity or created a more corrosion-prone surface. Lastly, the IPC group displayed the most negative ECORR (−495 ± 54 mV), indicating increased corrosive activity. All values were statistically significant (*p* < 0.05). In summary, both ICORR and ECORR indicate that the C group had better corrosion resistance than the IP and IPC groups. The IPC group demonstrated the lowest resistance.

Various physicochemical factors influence corrosion resistance in this study, the most relevant being the formation of a TiO_2_ passive layer on the C group’s surface, which acts as a protective barrier against degradation. However, in the IP group, implantoplasty-induced mechanical alterations may compromise the integrity of this layer, facilitating corrosion onset. In the IPC group, bacterial contamination worsens this effect by creating a highly aggressive microbial environment. Microorganisms such as Staphylococcus aureus and *Porphyromonas gingivalis* can degrade the titanium oxide layer by producing acidic metabolites and inducing localised electrochemical reactions, accelerating corrosion and increasing material deterioration. This suggests that implantoplasty and bacterial contamination have a negative synergistic effect on the electrochemical stability of titanium dental implants.

**Figure 1:**
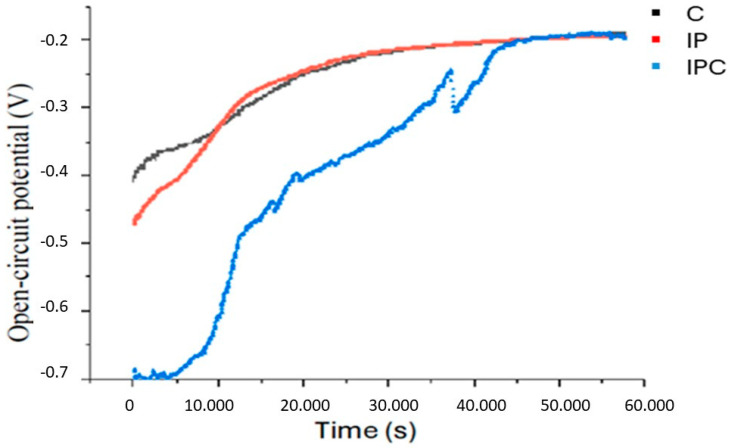
Open-circuit potential graph.

Figure 1 illustrates the evolution of the open circuit potential (EOCP) over time for the three studied groups. The C group shows the most positive values from the beginning and follows an upward trend until stabilising around −0.2 V, indicating greater corrosion resistance and a more positive and stable EOCP. The IP group exhibits a similar trend to the C group, albeit with slightly more negative values at the initial stages. This suggests that implantoplasty may affect the initial stability of the material; however, over time, the potential stabilizes and reaches values comparable to those of the control group. The IPC group, on the other hand, shows significantly more negative values from the start and a more unstable evolution. Its recovery is slower, and the potential remains within the range of −0.7 V to −0.3 V, indicating lower electrochemical stability. These findings suggest that the combination of implantoplasty and bacterial activity increases susceptibility to corrosion.

**Figure 2:**
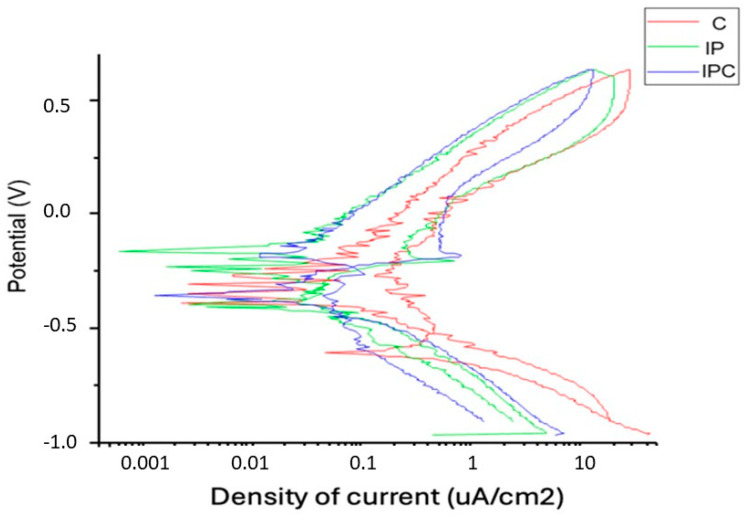
Current density graph.

Figure 2 presents potentiodynamic polarisation curves for the three studied groups. The current density (μA/cm^2^) represents the amount of current flowing per unit area of the implant, where higher values indicate a greater corrosion rate. The potential (V) represents the electrode potential of the material in the medium, with more negative values indicating higher susceptibility to corrosion. The shape of each curve reflects the electrochemical behaviour of the material under different conditions. The C group displays the most positive ECORR values and the lowest current density (ICORR), indicating a more stable passivation zone with minimal fluctuations, suggesting greater passive stability and a lower corrosion rate. The IP group exhibits slightly more negative potential, a higher ICORR, and greater irregularities in the passivation zone, suggesting that implantoplasty increases the material’s corrosive activity. The IPC group shows the most negative ECORR and the highest ICORR, with the least stable passivation zone, indicating a higher active corrosion state, likely due to the combined effects of implantoplasty and bacterial contamination.

### 3.2. Fatigue Tests

**Figure 3:**
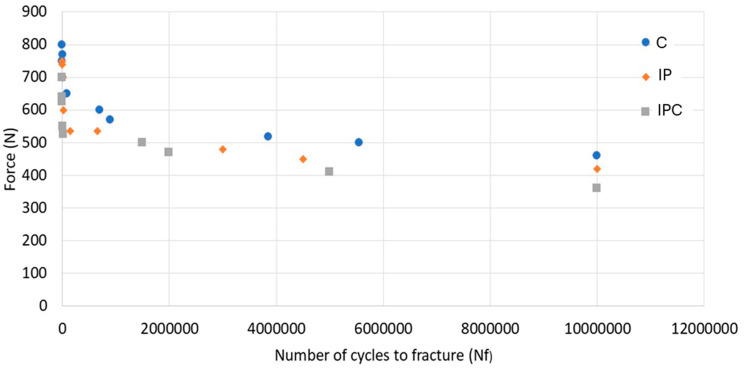
Fatigue curve (S-N) obtained for samples from groups C, IP, and IPC. NF: number of load cycles, N: force expressed in Newtons.

Figure 3 presents a graph illustrating the relationship between the applied force, represented in Newtons (N), and the number of cycles (Nf) in the three studied groups. The data show a dispersion pattern, indicating variability in fatigue resistance among the groups. The control group (C) exhibited the highest fatigue resistance, enduring more cycles before failure under the same applied force. In contrast, the IP group showed a lower resistance, with more dispersed data and a tendency to fail earlier than the control group. The IPC group displayed the lowest fatigue resistance, failing after fewer cycles, as evidenced by the leftward shift of its data points in the graph.

Three consecutive samples from the IPC group withstood 10 × 10^6^ load cycles at an initial load of 360 N without showing apparent damage. In contrast, three consecutive samples from the IP group demonstrated a fatigue limit of 420 N, while the control group reached a fatigue limit of 460 N.

Thus, the IPC group exhibited lower fatigue resistance compared to the IP and control groups. A general trend was observed across all groups: the force required to cause fracture decreased as the number of cycles increased. At lower forces (below 500 N), the implants could endure more cycles before fracturing, reaching up to 10 million cycles.

Specifically, a 21.7% reduction in fatigue resistance was observed in the IPC group compared to the control group. This suggests that implantoplasty weakens mechanical resistance by thinning the implant walls and generating residual stress. Additionally, bacterial contamination amplifies this effect by inducing localised corrosion (pitting), which promotes crack formation and further reduces fatigue resistance.

### 3.3. SEM Images Analysis

The samples were evaluated using scanning electron microscopy (SEM).

**Figure 4:**
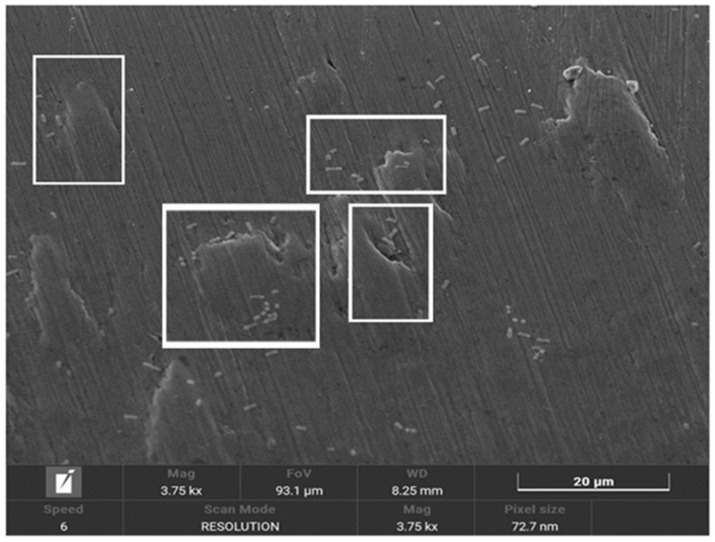
Scanning electron microscopy image of the surface of implants from the IPC group.

**Figure 5:**
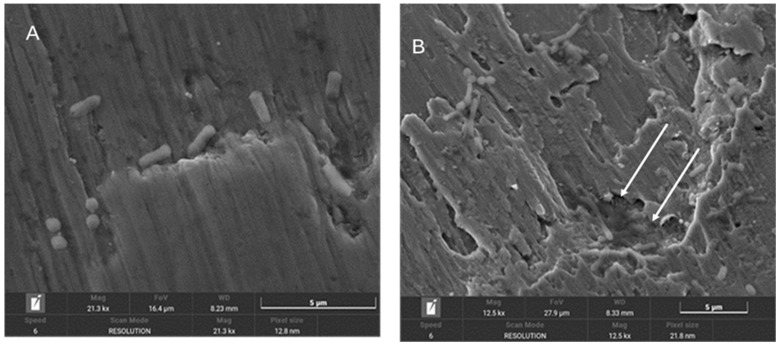
(**A**) SEM images of the surface of implants from the IPC group. (**B**) Image of the same area after 48 h.

In Figure 4, the presence of bacteria seeded on the surface of implants treated with implantoplasty can be observed, with the bacteria preferentially localising to the machined areas or regions with defects. Figure 5 shows bacteria surrounding a titanium defect and the same area after 48 h, where an increased number of bacteria and significant surface damage to the implant are evident. Additionally, the formation of a biofilm on the titanium surface can be observed.

**Figure 6:**
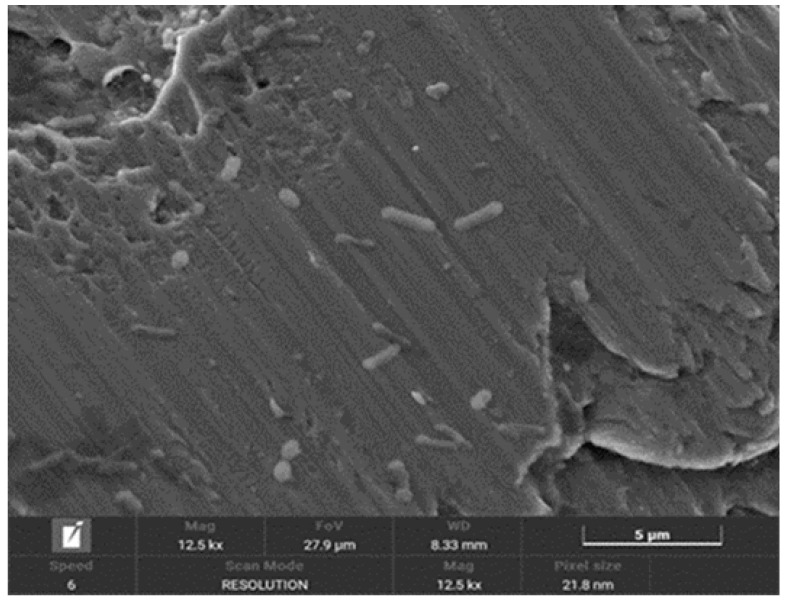
SEM image showing the effects of degradation caused by bacterial activity.

**Figure 7:**
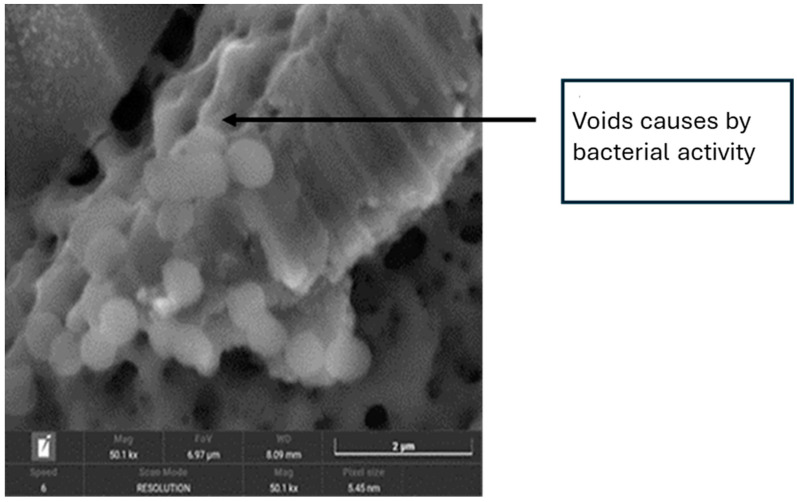
SEM image showing a crack caused by bacterial colonisation.

**Figure 8:**
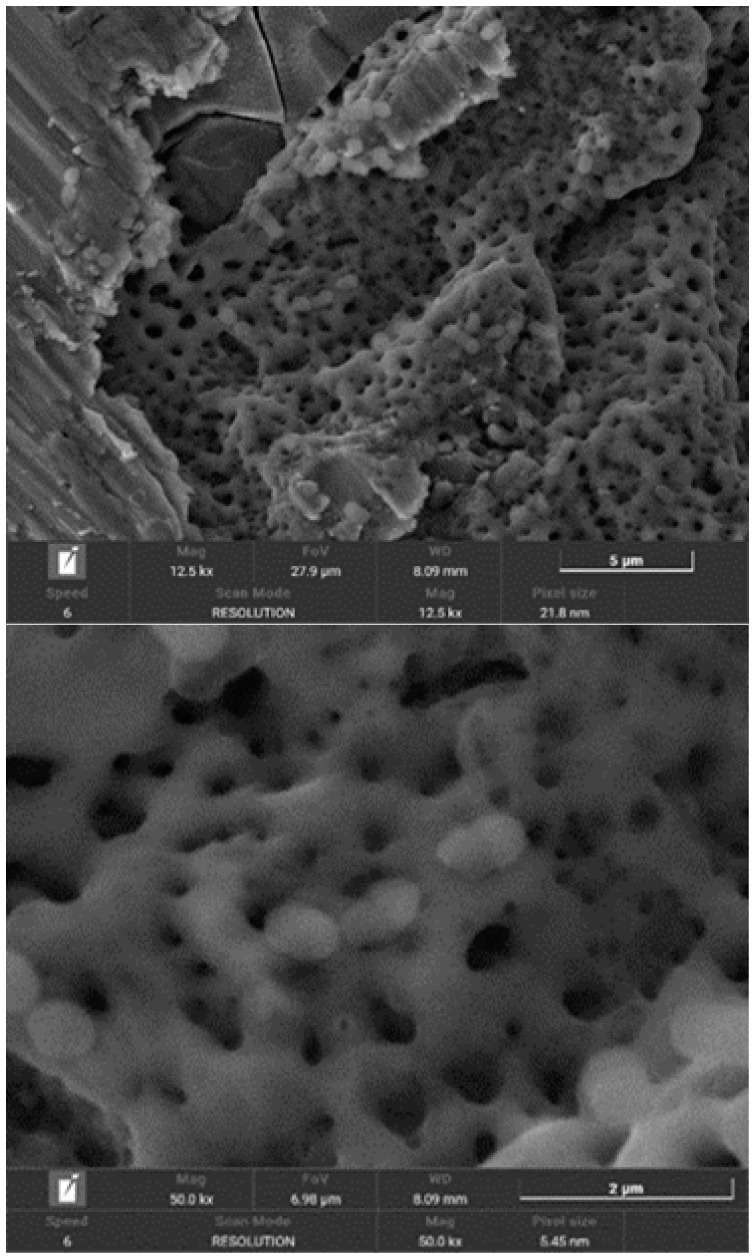
SEM images of the surface of implants from the CI group (implantoplasty and bacterial contamination).

Figure 6 illustrates the effects of bacterial-induced degradation, while Figure 7 displays a crack formed as a result of bacterial colonisation. Figure 8 shows the surface of the implants from the CI group (implantoplasty and bacterial contamination). These defects act as stress concentrators and facilitate crack initiation on the implantoplasty-treated surface, thus explaining the reduced fatigue life of the dental implant.

## 4. Discussion

This study evaluated the mechanical degradation of implant surfaces subjected to implantoplasty and contaminated with various bacterial strains, highlighting the impact of tribocorrosion, to simulate in vitro conditions that may occur clinically. To date, no studies have assessed the impact of bacteria on implants treated with implantoplasty.

The results demonstrated that under in vitro conditions, the presence of bacteria reduced corrosion resistance, evidenced by the formation of pitting on titanium surfaces. Furthermore, the interaction between mechanical forces generated during implantoplasty and bacteria-induced corrosion significantly decreased the implant’s fatigue resistance.

It is important to highlight that the oral environment is particularly hostile for metals. Various factors influence the tribocorrosion of dental implants in the oral cavity, such as the type of alloy, the presence of biofilm, low pH, galvanic interactions, masticatory forces, the composition of saliva, and its fluoride concentration [2]. These factors can alter the results compared to studies conducted in vitro.

The exposure to metabolic products of microorganisms within a biofilm causes microbial corrosion and surface discoloration, altering the oxidation state of titanium [32]. These findings suggest that titanium dissolution products may be associated with peri-implantitis and could modify the peri-implant microbiome [12,13,33].

Bacteria such as Staphylococcus aureus and *Porphyromonas gingivalis*, both used in this study, are commonly associated with peri-implantitis, inflammatory responses, and bone loss [27]. Although implantoplasty reduced surface roughness in two of our groups, bacterial adhesion and subsequent recolonisation were not prevented. This suggests that while smoother surfaces may hinder biofilm formation, implantoplasty alone is insufficient to eliminate bacterial colonisation [34].

Bacterial corrosion is an electrochemical process facilitated by bacteria in the presence of water, generating redox reactions that produce volatile sulfur compounds and electric currents [11,35]. These currents enable electron transfer between the metal and bacteria, accelerating brittleness and crack propagation, especially in areas with stress differences and surface topography variations [8,36]. In this study, the interface between rough and implantoplasty-treated areas exhibited higher internal energy, promoting electrochemical corrosion and increasing corrosion rates, particularly at the smooth-to-rough interface [23,37], accelerating brittleness and crack formation in the material [9]. These results align with previous studies showing how bacterial presence reduces titanium’s corrosion resistance by attacking and thinning its passive TiO_2_ layer [1,38].

Scanning electron microscopy (SEM) images from our study confirm significant bacterial presence and extensive surface damage on the implant.

Implantoplasty, while reducing plaque retention, lowers corrosion resistance and fracture resistance due to wall thinning [39]. This process generates high residual stress, promoting electrochemical corrosion, pitting corrosion, discoloration, and the release of metallic ions into saliva. These factors, combined with bacterial products and chloride ions, accelerate metal degradation [8,40,41]. In our study, a significant reduction in corrosion resistance was observed (*p* < 0.05). The control group exhibited the highest corrosion resistance, followed by the implantoplasty group and then the implantoplasty with bacterial contamination group. Although EOCP values between the latter two groups were not significantly different, significant differences in ECORR and ICORR values indicated that bacterial contamination increased corrosion susceptibility. In line with the results of Lozano et al. [7] and Camps-Font et al. [19], it is observed that implantoplasty thins the walls of the implants, generating high residual stress that promotes electrochemical corrosion. This supports the reduction in corrosion resistance in implants treated with implantoplasty. However, these studies do not take bacterial contamination into account.

The fatigue resistance of materials is essential for predicting their long-term performance. In a corrosive environment such as the oral cavity, fatigue resistance decreases, increasing the risk of implant failure after fewer load cycles. Compression cycles applied in this study replicated mastication forces exceeding typical physiological loads. Previous studies have shown varying impacts of implantoplasty on fatigue resistance, with some reporting no significant reduction [19], while others observed reductions up to 30% [26]. Despite this variability, it is widely accepted that forces exceeding 500 N surpass physiological thresholds, ensuring an infinite lifespan for implants under normal conditions [22,37]. However, our findings indicate that implantoplasty, especially when combined with bacterial contamination, reduces fatigue resistance. The control group exhibited the longest lifespan with a fatigue limit of 460 N, while the implantoplasty group reached 420 N, and the bacterial contamination group only reached 360 N.

Figure 4, Figure 5 and Figure 6 show in the fractographies the crack advancement lines corresponding to the crack advancement in the fatigue process. In the same way, we can observe that the deformations produced in the titanium by the advance of the crack are rounded due to the chemical attack by the physiological liquids, similar to a chemical attack [4,42]. This corrosive effect accelerates the crack propagation, reducing the fatigue life of the implant. Likewise, it can be seen how the areas of greater bacterial colonisation are in the places where there has been greater plastic deformation, in the areas of the burrs or defects produced by the drills. This occurs since the areas of greater mechanical stress, i.e., the areas with greater internal energy, are the areas most susceptible to bacterial colonisation, since this energy is used by bacteria. This fact has been observed, as the titanium crystal boundaries are also the areas of highest bacterial proliferation due to the energy of the grain boundaries [43,44].

A marginal bone loss of 6 mm was simulated in this study, representing an extreme clinical scenario. This reduced insertion level increased stress on peri-implant tissues, consistent with findings from previous studies showing higher fracture risk in cases of significant bone loss. Although ISO 14801:2016 recommends a reduction of 2.5 to 3.5 mm, greater bone loss is often observed clinically due to peri-implantitis, necessitating treatments like implantoplasty. However, this approach increases risks, such as localised corrosion and accelerated material degradation [45].

This study has several limitations. Only two bacterial strains were included, despite the complexity of the peri-implant biofilm, which consists of multiple bacterial species. Additionally, a single type of implant surface was used, even though commercially pure titanium implants exhibit variable mechanical properties. Future research should consider dynamic bacterial biofilms and different implant surfaces to expand on the findings presented here.

## 5. Conclusions

In conclusion, this study demonstrated that implantoplasty and bacterial contamination significantly reduce the corrosion and fatigue resistance of dental implants. Specifically, the corrosion current density increased from low values in the C group (0.025 ± 0.011 μA/cm^2^) to higher values in the IPC group (0.122 ± 0.030 μA/cm^2^), indicating a decrease in corrosion resistance. Regarding fatigue resistance, the IPC group showed a 21.7% reduction compared to the control group.

Implants subjected to implantoplasty and bacterial contamination exhibited a notable decrease in mechanical resistance due to pitting corrosion and degradation induced by bacterial metabolic products. Furthermore, the results highlight the importance of considering the bacterial effects on the durability of implants treated with implantoplasty, as bacterial recolonisation occurs rapidly.

## Figures and Tables

**Table 1:** Electrochemical and corrosion parameters evaluated for dental implants.

	Grupo C	Grupo IP	Grupo IPC	*p*-Value
EOCP (mV)	−137.7 ± 5.2	−194.3 ± 3.6	−198.23 ± 5.7	*p* < 0.001
ICORR (μA/cm^2^)	0.025 ± 0.011	0.089 ± 0.022	0.122 ± 0.030	*p* < 0.001
ECORR (mV)	−380 ± 18	−450 ± 40	−495 3 ± 54	*p* < 0.001

## Data Availability

The original contributions presented in the study are included in the article. Further inquiries can be directed to the corresponding authors.
